# Refractory Kawasaki Disease With a Coronary Artery Aneurysm in a Three-Month-Old Infant: Diagnostic Challenges and Rescue With Infliximab

**DOI:** 10.7759/cureus.100747

**Published:** 2026-01-04

**Authors:** Diyar H Nernji, Emad Elhuni, Ruqaiya Al Jashmi, Ahlam Atiq

**Affiliations:** 1 Pediatric Critical Care, Sultan Qaboos Hospital, Salalah, OMN; 2 Pediatric Hematology, Sultan Qaboos Hospital, Salalah, OMN; 3 Pediatric Rheumatology, Royal Hospital, Muscat, OMN; 4 Pediatric Cardiology, Sultan Qaboos Hospital, Salalah, OMN

**Keywords:** coronary aneurysm, echocardiography, infliximab, refractory kawasaki disease, vaculitis

## Abstract

Kawasaki disease (KD) is an acute systemic vasculitis and the leading cause of acquired heart disease in children. Diagnosis is particularly challenging in early infancy due to incomplete presentations. We describe a three-month-old infant with persistent fever, irritability, and lymphadenopathy who was initially treated for bacterial infection.

By Day 7, the patient developed conjunctivitis, cracked lips, and pharyngeal congestion, meeting criteria for incomplete Kawasaki disease. Laboratory findings included leukocytosis, anemia, thrombocytosis, elevated C-reactive protein, and hypoalbuminemia. Initial echocardiography was normal; intravenous immunoglobulin (2 g/kg) with aspirin led to temporary improvement.

Fever recurred, and repeat imaging revealed a right coronary artery aneurysm. Despite additional immunoglobulin and corticosteroids, the patient improved only after infliximab, with regression of coronary changes.

This report emphasizes the diagnostic challenges of incomplete KD in early infancy, demonstrates practical application of the 2017 American Heart Association (AHA) diagnostic algorithm, and highlights the role of timely escalation to biologic therapy in refractory cases.

## Introduction

Kawasaki disease (KD) is an acute systemic vasculitis primarily affecting children under five years of age and remains the leading cause of acquired heart disease in childhood. Without timely treatment, approximately 25% of patients develop coronary artery (CA) dilatation or aneurysms (CAAs), emphasizing the crucial importance of early recognition and management [1].

The incidence of KD varies geographically: in the United States, it affects 18-25 per 100,000 children under five years annually, compared with a 10- to 30-fold higher rate in Northeast Asian countries, including Japan, South Korea, China, and Taiwan [1,2]. Diagnosis is based on established clinical criteria, including a persistent high-grade fever for five or more days plus four or more of the following features: bilateral non-purulent conjunctivitis, mucosal changes (e.g., strawberry tongue, cracked lips), polymorphous rash, extremity changes (edema or peeling of the skin), and cervical lymphadenopathy of at least 1.5 cm in diameter [1].

However, a significant proportion of patients, particularly infants, present with incomplete or atypical Kawasaki disease, lacking the classical diagnostic criteria. In these cases, supportive laboratory findings and echocardiographic evidence of coronary involvement become essential for timely diagnosis. Key laboratory indicators include elevated inflammatory markers (e.g., CRP, ESR), normocytic anemia, hypoalbuminemia, and sterile pyuria [1].

Infants ≤ 6 months of age often present with prolonged fever lacking other classic features of Kawasaki disease and are at higher risk of coronary artery abnormalities. Diagnostic delays are common in this age group, further increasing the likelihood of coronary involvement [3].

Coronary artery aneurysms (CAAs) represent the most serious complication of Kawasaki disease. The main risk factors include prolonged fever, delayed initiation of therapy, male sex, markedly elevated inflammatory factors (C-reactive protein or erythrocyte sedimentation rate), and failure to respond to initial intravenous immunoglobulin (IVIG) therapy [4]. The risk of CAAs increases significantly in young infants: approximately 40% in infants aged 6-12 months and up to 68% in those under six months of age [5]. Nearly 50% of infants <6 months have a baseline coronary artery Z-score ≥2.5 on initial echocardiography [6,7]. Son et al. developed a North American risk score including age <6 months, Asian race, initial CA Z-score >2, and CRP >130 mg/L, with a score ≥3 strongly predicting CAA by eight weeks [8].

First-line treatment consists of high-dose IVIG (2 g/kg) and aspirin, while corticosteroids or monoclonal antibodies such as infliximab are used for IVIG-resistant cases [9]. Emerging evidence supports the use of interleukin-1 blockers (e.g., anakinra) due to upregulation of IL-1 pathway genes in the acute inflammatory phase of Kawasaki disease [10].

## Case presentation

A three-month-old infant with no prior health issues and normal developmental milestones presented to the hospital with a one-day history of high-grade, unremitting fever and irritability, associated with decreased oral intake. He had mild upper respiratory symptoms - including cough and rhinorrhea - for several days, but denied gastrointestinal symptoms or abnormal movements. His left cheek had a non-pruritic rash for approximately one month. There was a reported history of recent household exposure to a family member with an upper respiratory tract infection. He was born at 36 weeks’ gestation via spontaneous vaginal delivery, large for gestational age, with a birth weight of 3.5 kg. His mother is diabetic. He was admitted to the neonatal intensive care unit for 48 hours for neonatal hyperbilirubinemia, requiring a single phototherapy session, and was discharged in stable condition.

Examination

On admission, the infant weighed 6.2 kg (75th percentile) and measured 63 cm in length (50th percentile). Vital signs were as follows: temperature 39.0°C, respiratory rate 50 breaths/min, heart rate 185 beats/min, oxygen saturation 99% on room air, and blood pressure 85/50 mmHg. He appeared ill and febrile but was non-dysmorphic. Ocular examination was unremarkable. There was an enlarged left submandibular lymph node, measuring >1.5 cm, firm, tender, and without overlying erythema. Oral examination revealed no mucosal changes initially. A circular scaly macular lesion was noted on the left cheek, clinically consistent with tinea corporis (ringworm). Neurological examination revealed an alert infant, but irritable with handling. The anterior fontanelle was mildly depressed, and no focal neurological deficits were identified. Cardiovascular examination revealed persistent sinus tachycardia, with no murmurs, gallops, or rubs. Peripheral pulses were well palpated, extremities were warm, and there were no clinical signs of shock. Respiratory auscultation was clear bilaterally. The abdomen was soft, non-tender, and without organomegaly. Joints, hands, and feet were unremarkable. Mild erythema of the perineum was noted. Despite initiation of empiric antimicrobial therapy with a third-generation cephalosporin and vancomycin, fever remained unremitting, and irritability persisted. The left submandibular lymphadenopathy progressively enlarged over the first seven days of admission. Tachycardia remained persistent. By Day 7 of illness, new mucocutaneous features developed: bilateral non-exudative conjunctival injection, cracked lips, mild congestion of the soft palate and pharynx (tongue remained normal, without strawberry appearance), and perineal erythema with desquamation. The hands and feet remained without oedema, rash, or peeling at this stage. Given the presence of multiple mucocutaneous features and persistent systemic inflammation, a diagnosis of incomplete Kawasaki disease was suspected - prompting initiation of immunomodulatory therapy.

Investigations

On the first day of admission, laboratory results showed neutrophilic leukocytosis (white blood cells 23 × 10⁹/L, neutrophils 53%), normocytic anaemia (haemoglobin 10 g/dL) with elevated platelet count 537 × 10⁹/L. The laboratory investigation also revealed mildly elevated C-reactive protein (CRP) of 31 mg/L. Renal function, serum electrolytes, liver function tests, and urinalysis were within normal limits. Electrocardiogram revealed sinus tachycardia with no ischemic changes. Neck ultrasonography demonstrated enlarged left submandibular and cervical lymph nodes without abscess. Chest radiography and abdominal ultrasound were unremarkable.

On Day 3 of admission, due to persistent fever, repeat tests were performed. Laboratory tests showed WBC 17 × 10⁹/L with neutrophils 12 × 10⁹/L, haemoglobin 8.5 g/dL (normocytic, normochromic), and platelet count 400 × 10⁹/L. Serum albumin was normal. Urine culture grew Proteus mirabilis (likely contaminant), while blood culture showed no growth. Repeated urine and blood cultures were obtained for surveillance. Cerebrospinal fluid (CSF) analysis revealed normal cell count, protein, and glucose.

On Day 7 of admission, WBC remained 17 × 10⁹/L with neutrophils 12 × 10⁹/L, haemoglobin had further decreased to 8.1 g/dL (normocytic, normochromic), and platelet count had risen to 800 × 10⁹/L. CRP increased markedly to 236 mg/L, and serum albumin had dropped to 31 g/L. Urinalysis revealed 20 WBCs/high-power field (HPF), and repeat urine and blood cultures showed no growth. Initial echocardiography revealed coronary artery dimensions within normal limits for body surface area with preserved biventricular systolic function and no pericardial effusion. 

A summary of the patient’s laboratory results over the first week of admission is shown in Table 1.

**Table 1:** Serial laboratory investigations of the three-month-old infant with refractory Kawasaki disease Laboratory values are presented by day of admission. Reference ranges are age-appropriate for a three-month-old infant. "Not repeated" indicates the test was not performed on that day. WBC: white blood cells; MCV: mean corpuscular volume; CRP: C-reactive protein; ALT: alanine aminotransferase; AST: aspartate aminotransferase; hpf: high-power field; CSF: cerebrospinal fluid

Test	Reference Range	Day 1	Day 3	Day 7
WBC (×10⁹/L)	4.0–12.0	23	17	17
Neutrophils (×10⁹/L)	1.5–8.5	12.2 (53%)	12	12
Hemoglobin (g/dL)	10.5–14.0	10	8.5	8.1
MCV (fL)	75–95	Normocytic	Normocytic	Normocytic
Platelets (×10⁹/L)	150–400	537	400	800
CRP (mg/L)	<5	31	Not repeated	236
Albumin (g/L)	35–50	Normal	Not repeated	31
ALT (U/L)	7–56	Normal	Not repeated	Not repeated
AST (U/L)	10–40	Normal	Not repeated	Not repeated
Creatinine (µmol/L)	18–35	Normal	Normal	Normal
Electrolytes	Normal	Normal	Normal	Normal
Urinalysis (WBC/hpf)	<5	Normal	Not repeated	20
Blood culture	—	No growth	No growth	No growth
Urine culture	—	Not repeated	Proteus mirabilis (likely contaminant)	No growth
CSF analysis	Normal	Not repeated	Normal	Not repeated

Treatment

Based on the initial clinical and laboratory findings (Days 1-6), the patient was diagnosed with an invasive bacterial infection and started on intravenous cefotaxime. Vancomycin was added on Day 3.

By Day 7 of admission, he developed cracked lips, non-purulent conjunctivitis, and perineal erythema, accompanied by rising inflammatory markers. A diagnosis of Kawasaki disease (KD) was made. Intravenous immunoglobulin (IVIG) 2 g/kg (single dose) and high-dose aspirin 50 mg/kg/day in four divided doses were initiated. Echocardiography at this stage showed normal coronary arteries. Fever subsided within 24 hours of IVIG, irritability resolved, tachycardia improved, and oral intake normalized. The patient remained afebrile for two days, and aspirin was reduced to 5 mg/kg/day on day 10 of illness. He was discharged with follow-up.

**Figure 1:**
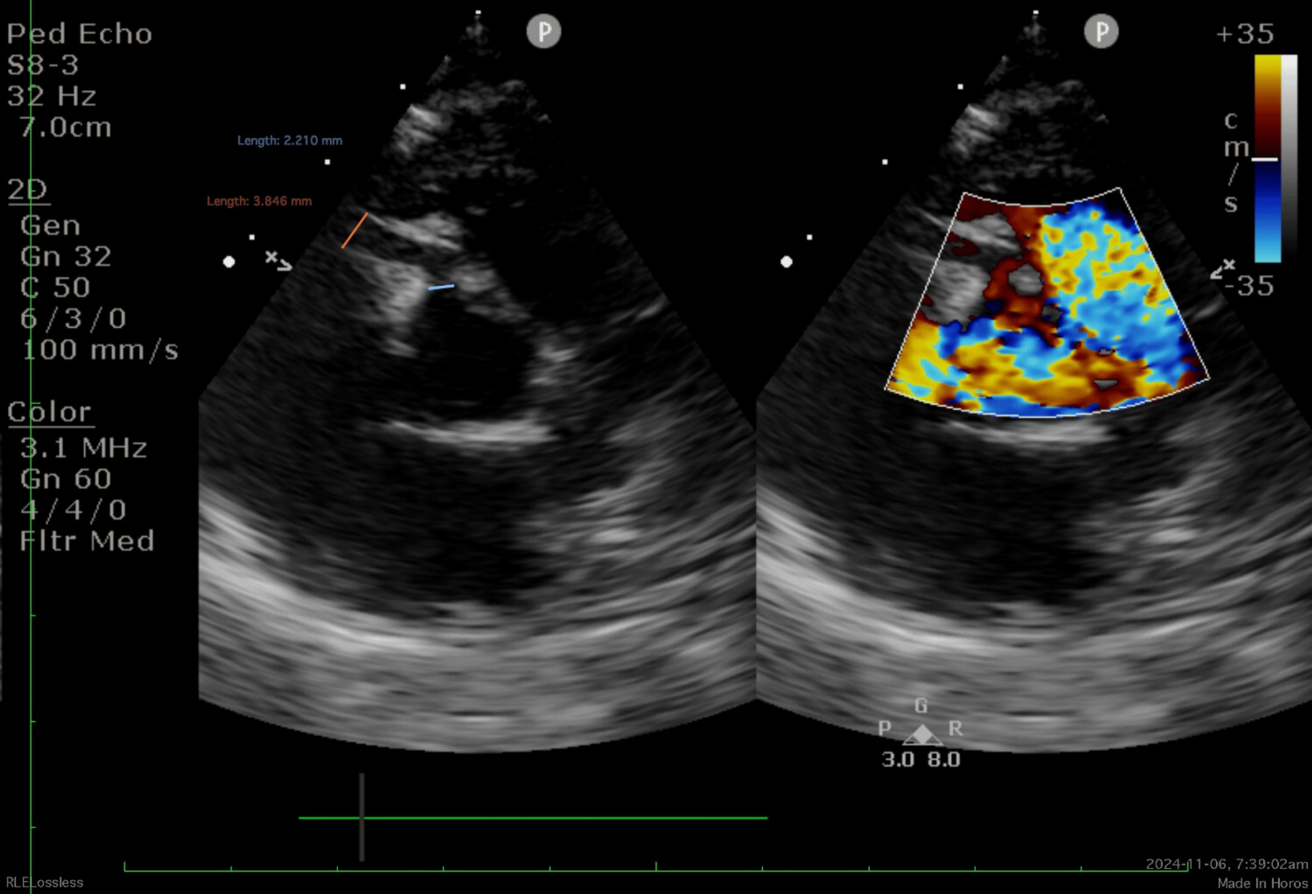
Echocardiography demonstrated proximal right coronary artery (RCA) dilatation measuring 2.2 mm (Z score +2.77) and an RCA aneurysm measuring 3.9 mm (Z score +7.93)

On Day 12 of illness, he was readmitted with recurrent fever (up to 38°C) and new cough and cold symptoms. Repeat echocardiography on Day 14 revealed proximal right coronary artery (RCA) dilatation (2.2mm; Z score +2.77), and RCA aneurysm (3.9 mm; Z score +7.93), normal left main coronary artery dimensions, and a mild posterior pericardial effusion (Figure 1). Coronary artery dimensions were reviewed, and z-scores were calculated using the Dallaire formula [11]. The diagnosis of refractory KD was established according to the 2017 American Heart Association (AHA) criteria [1]. After multidisciplinary discussion, treatment included a second dose of IVIG (2 g/kg), intravenous methylprednisolone (10 mg/kg/day for five days) followed by tapering oral prednisolone, and prophylactic low molecular weight heparin (50 IU/kg twice daily) to reduce thrombosis risk. Fever mildly subsided, and the patient remained stable.

**Figure 2:**
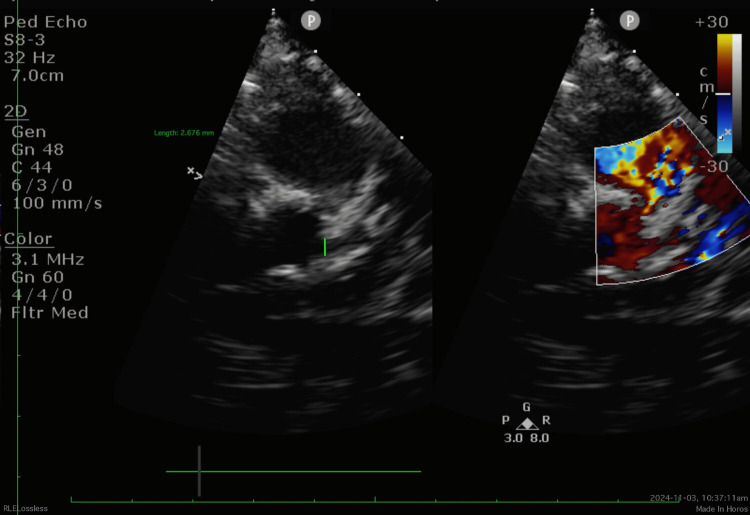
Echocardiography showed diffuse left main coronary artery (LMCA) dilatation measuring 2.7 mm (Z score +3.65)

Follow-up echocardiography on Day 17 showed a diffuse left main coronary artery (LMCA) dilatation (2.7 mm; Z score +3.65), persistent RCA aneurysm, preserved cardiac function, and mild pericardial effusion (Figure 2). Given these findings, infliximab (6 mg/kg infusion) was administered with pre-medications (chlorpheniramine and paracetamol). The infusion was well tolerated.

On Day 20, echocardiography demonstrated improvement: LMCA diameter decreased to 2.2 mm (Z score +2.12), proximal RCA measured 2.1 mm (Z score +2.47), and RCA aneurysm regressed to 3.2 mm (Z score +5.81). Cardiac function remained preserved with no mitral regurgitation, trace tricuspid regurgitation, and persistent mild pericardial effusion. CRP had declined to 1 mg/L.

**Figure 3:**
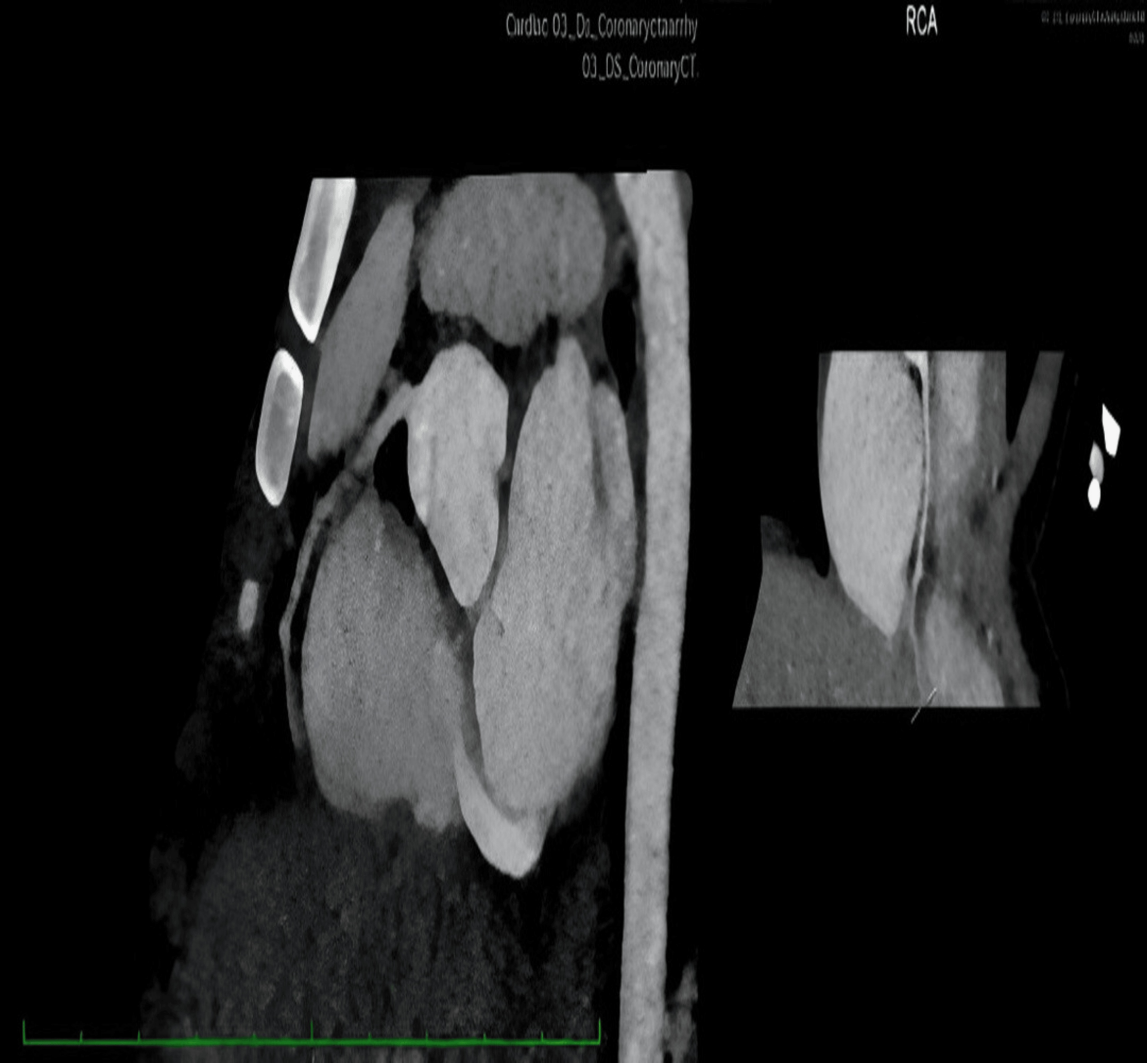
Computed tomography coronary angiography (CTCA) demonstrated regression of coronary arteries dilatation and resolved RCA aneurysm.

The patient was discharged on a tapering dose of oral prednisolone and low-dose aspirin (5 mg/kg/day) to be continued for 12 months. Low-molecular-weight heparin was discontinued before discharge. He remained clinically well during outpatient follow-up visits. Echocardiography performed one month post-discharge showed resolution of the (RCA) aneurysm, with the RCA measuring 1.53 mm and no evidence of aneurysmal dilatation. At seven months after illness onset, computed tomography coronary angiography (CTCA) performed at a tertiary cardiac centre demonstrated regression of the coronary artery dilatation, and the previously reported RCA aneurysm had completely resolved (Figure 3). 

The patient’s clinical course, laboratory findings, echocardiographic changes, treatments, and responses over time are summarized in Table 2.

**Table 2:** Summary of the clinical course, laboratory findings, echocardiographic features, treatment, and outcomes in our case of refractory Kawasaki disease. Abbreviations: WBC, white blood cell count; Hb, hemoglobin; Plt, platelet count; CRP, C-reactive protein; KD, Kawasaki disease; IV, intravenous; IVIG, intravenous immunoglobulin; RCA, right coronary artery; LMCA, left main coronary artery; LMWH, low-molecular-weight heparin; CTCA, computed tomography coronary angiography.

Time point	Clinical features	Lab findings	Echo Findings	Treatment	Response
Day 1	High-grade fever, irritability, submandibular lymphadenopathy, perineal erythema	WBC:23×10⁹/L Hb 10 g/dL, Plt: 537×10⁹/L, CRP: 31 mg/L	Not done	IV cefotaxime	Fever persists
Day 3	Persistent fever, irritability	WBC 17×10⁹/L, Hb 8.5 g/dL, Plt 400×10⁹/L	Not done	Added IV vancomycin	No improvement
Day 7	Bilateral conjunctival injection, cracked lips, perineal erythema	WBC 17×10⁹/L, Hb 8.1 g/dL, Plt 800×10⁹/L, CRP 236 mg/L, albumin 31 g/L Blood and urine cultures: No growth.	Normal	Diagnosis: incomplete KD→ IVIG 2 g/kg, high-dose aspirin	Fever subsided within 24h, irritability improved
Day 10	Afebrile, improved oral intake	Not done	Not done	Aspirin reduced to 5 mg/kg/day	Discharged
Day 12	Recurrent fever, cough	Not done	Not done	Readmission	—
Day 14	Persistent fever	Not done	RCA aneurysm (3.9 mm; Z score +7.93), LMCA normal.	2nd IVIG 2 g/kg, IV methylprednisolone 10 mg/kg/day ×5, LMWH	Fever mildly subsided
Day 17	Afebrile	Not done	LMCA 2.7 mm; Z score +3.65, RCA persistent aneurysm	Infliximab 6 mg/kg	Improvement begins
Day 20	Clinically stable	CRP 1 mg/L	LMCA 2.2 mm, RCA aneurysm 3.2 mm; Z score +5.8	Tapered prednisolone, aspirin continued	Coronary improvement
1 month	Clinically well	Not done	RCA 1.53 mm, no aneurysm	Tapered prednisolone, aspirin continued	Stable
7 months	Clinically well	Not done	CTCA: complete regression of the coronary artery dilatation	Treatment stopped	Fully resolved

## Discussion

Kawasaki disease in early infancy poses significant diagnostic limitations. Clinical manifestations are often incomplete and evolve over time, overlapping with common infections such as bacterial lymphadenitis or viral exanthems [1].

The differential diagnosis includes infectious causes such as adenovirus (can be distinguished by PCR), scarlet fever, measles, and enterovirus; inflammatory conditions like multisystem inflammatory syndrome in children (MIS-C) with elevated troponin, juvenile idiopathic arthritis, and rheumatic fever (both are unusual in this age group) [1].

Kawasaki disease (KD) should be considered in any child with unexplained fever persisting for five or more days, particularly if accompanied by conjunctivitis, oral changes, extremity changes, rash, or lymphadenopathy [12]. Infants under six months of age have up to a 35% risk of developing coronary artery aneurysms despite timely IVIG treatment, while children aged 6-12 months also have an increased risk of IVIG resistance [1,6,13,14].

Refractory KD, defined as persistent or recurrent fever 36 hours after completion of initial intravenous immunoglobulin (IVIG) therapy or evidence of progressive coronary involvement, occurs in up to 15% of cases [15]. Persistent fever is a major predictor of coronary complications [16]. Laboratory findings - such as anemia, thrombocytosis, hypoalbuminemia, and elevated inflammatory markers - can support the diagnosis in incomplete cases [1,17].

In this case, the early presentation with fever and isolated lymphadenopathy contributed to diagnostic confusion and an initial assumption of bacterial infection. The delayed appearance of mucocutaneous features postponed consideration of KD until the seventh day of illness, despite persistent inflammation. Application of the 2017 American Heart Association (AHA) diagnostic algorithm later supported the diagnosis, yet the normal initial echocardiogram and non-specific laboratory findings contributed to diagnostic uncertainty. These factors illustrate the difficulty of early recognition and the potential for coronary injury even with apparently timely therapy. Recent reports from the post-COVID-19 era suggest that increasing cardiac complications may, in part, reflect delayed or missed recognition of KD, underscoring the importance of heightened clinical vigilance [18].

In this case, relapse with fever and the appearance of a right coronary artery aneurysm fulfilled the criteria for refractory Kawasaki disease. A second IVIG dose and corticosteroids failed, but infliximab achieved clinical recovery and regression of coronary changes. Infliximab, an anti-TNF-α monoclonal antibody, has been shown to rapidly reduce fever and systemic inflammation in IVIG-resistant KD [9,19-22]. Other adjunctive therapies, including cyclosporine and interleukin inhibitors (e.g., anakinra, tocilizumab), may be used in selected high-risk or refractory cases based on clinical response and risk stratification [1].

Infliximab, a monoclonal antibody, neutralizes elevated TNF-α implicated in Kawasaki disease pathogenesis, thereby mitigating endothelial damage of coronary arteritis. It’s indicated for IVIG-resistant cases (persistent fever >36 hours post-IVIG) or primary intensification. It is administered as a 6 mg/kg single IV infusion over two hours, optimally within 7-10 days of fever onset. Randomized controlled trials demonstrate reduced treatment resistance, shorter fever duration, and greater improvement in coronary artery dilatation at Week 2, with an excellent safety profile devoid of serious adverse events [9].

Choice of rescue therapy may be influenced by institutional resources and regional practice patterns, as highlighted in recent multinational pharmacoeconomic analyses [23].

## Conclusions

This case illustrates the diagnostic challenges of KD in infants under six months, who frequently present with incomplete or evolving clinical features that delay recognition. Early application of the 2017 American Heart Association (AHA) diagnostic algorithm, combined with careful interpretation of laboratory markers and repeat echocardiography, is critical in evaluating prolonged unexplained fever in this high-risk age group. Persistent or recurrent fever after initial intravenous immunoglobulin (IVIG) therapy should raise concern for refractory KD, as it strongly predicts coronary involvement. In this patient, timely escalation to infliximab after failure of second-line therapies resulted in rapid clinical improvement and regression of coronary artery changes. This case reinforces the importance of structured evaluation, vigilant monitoring, and prompt initiation of adjunctive therapies to optimize outcomes and reduce the risk of coronary complications in young infants with KD.
